# The role and application of small extracellular vesicles in glioma

**DOI:** 10.1186/s12935-024-03389-z

**Published:** 2024-06-29

**Authors:** Zhihao Yang, HaoYuan Wu, ZhiWei Wang, ErBao Bian, Bing Zhao

**Affiliations:** 1 Department of Neurosurgery, The Second Affiliated Hospital of Anhui Medical University, Hefei, 230601 Anhui Province China; 2 Cerebral Vascular Disease Research Center, Anhui Medical University, Hefei, 230601 Anhui Province China

**Keywords:** Small extracellular vesicles, Exosomes, Glioma

## Abstract

Small extracellular vesicles (sEVs) are cell-derived, nanometer-sized particles enclosed by a lipid bilayer. All kinds of biological molecules, including proteins, DNA fragments, RNA, lipids, and metabolites, can be selectively loaded into sEVs and transmitted to recipient cells that are near and distant. Growing shreds of evidence show the significant biological function and the clinical significance of sEVs in cancers. Numerous recent studies have validated that sEVs play an important role in tumor progression and can be utilized to diagnose, stage, grading, and monitor early tumors. In addition, sEVs have also served as drug delivery nanocarriers and cancer vaccines. Although it is still infancy, the field of basic and translational research based on sEVs has grown rapidly. In this review, we summarize the latest research on sEVs in gliomas, including their role in the malignant biological function of gliomas, and the potential of sEVs in non-invasive diagnostic and therapeutic approaches, i.e., as nanocarriers for drug or gene delivery and cancer vaccines.

**Supplementary Information:**

The online version contains supplementary material available at 10.1186/s12935-024-03389-z.

## Introduction

Glioma, the most common primary tumor of the central nervous system (CNS), is classified into four grades. Among them, the grade IV glioma, also called glioblastoma (GBM), is the most malignant and characterized by high invasiveness. Worldwide, there are about 100,000 people diagnosed with glioma every year, and GBM accounts for 70–75% of all diffuse glioma diagnoses [1, 2]. However, the prognosis of GBM patients is unsatisfactory, and the average survival is only 12–15 months. Moreover, in cancer patients between 15 and 34 years, GBM is the third most common cause of death [3, 4]. At present, the standard treatment of GBM is surgery and subsequent chemoradiotherapy. However, due to high heterogeneity and invasiveness, the outcome of GBM patients is still dismal [5–7]. Thus, in order to improve the survival rate and quality of life of glioma patients, more effective treatments and highly accurate and noninvasive tumor biomarkers which can identify the glioma even in its early stages are urgently needed.

sEVs released by most cells are essential to intercellular connections and pathophysiology. In recent years, research on sEVs has grown due to their unique structure and function. Previous studies showed that sEVs can transfer proteins and nucleic acids from the original cell to the target cells and then have biological effects on them. Regarding tumors themselves, growing evidence suggests that sEVs deriving from tumors participate in many physiological and pathological processes, including tumor microenvironment (TME), angiogenesis, epithelial-to-mesenchymal transition (EMT), immune regulation, metastasis, and therapeutic resistance [8–10].

Recently, many studies have verified that sEVs can be utilized for early diagnosis, staging, grading, and treatment monitoring of cancers [11, 12]. In addition, sEVs show potential as nanocarriers for cancer vaccines and drug delivery systems. sEVs have the advantages of specificity, safety, stability, and ability to penetrate the blood–brain barrier. These findings raise important clinical implications and highlight the need to further explore sEVs-based diagnostic and therapeutic approaches, especially in gliomas.

In this review, we primarily discuss the effects of sEVs on glioma progression, angiogenesis, metastasis, TME, resistance to treatment, liquid biopsy, and treatment. We hope to inspire readers to further explore the role of sEVs in glioma and that leveraging these properties may open new avenues to address the complexities of glioma diagnosis and treatment.

## The origin and characteristics of extracellular vesicle (EVs)

**Fig. 1:**
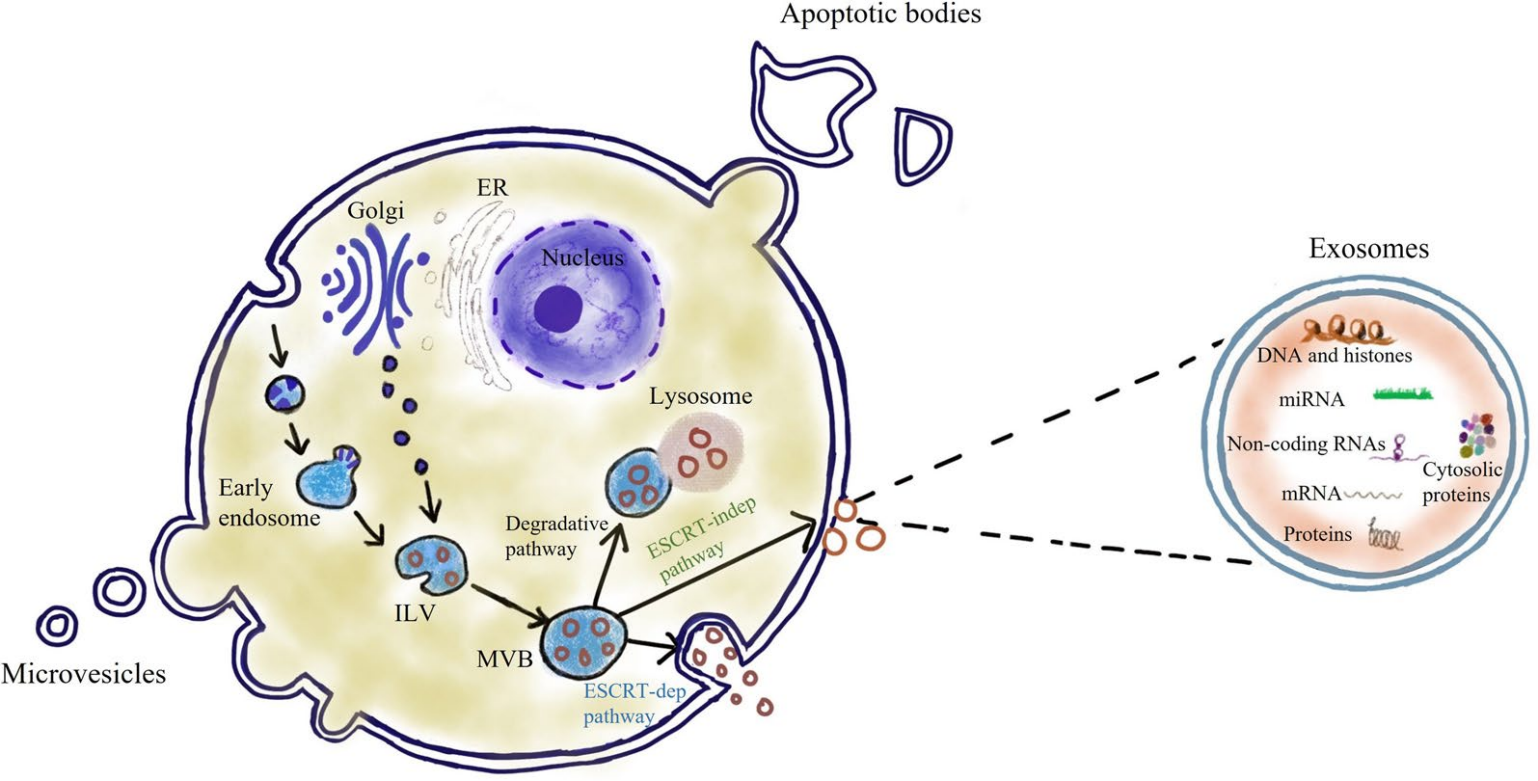
The biogenesis and cargo loading of sEVs. Cells mainly secrete three types of EVs, including microvesicles, apoptotic bodies and exsomes. Exsomes are formed in the endosomal compartment of the cell by inward budding of limiting endosome membrane into exosomal precursors, intraluminal vesicles (ILVs). Endosomal multivesicular bodies (MVBs) containing accumulated ILVs can then be degraded by fusing with lysosomes or autophagosomes or released to extracellular matrix as exosomes via ESCRT-independent or ESCRT-dependent mechanisms. The main contents of exosomes include miRNA, non-coding RNA, protein, etc

In 1983, John Stone et al. researched the transformation of sheep reticulocytes to mature erythrocytes and observed that the erythrocytes release transferrin metabolites by secreting small vesicles that were later called exosomes [13]. The secretion pattern is shown in Fig. 1. First, the plasma membrane buds inward and generates early endosomes, which are then processed into multivesicular bodies (MVBs) [14]. MVBs involve in endocytosis and transporting intracellular substances. There are two endings of MVBs. The lysosome degrades one. The other is fusing with the cell membrane and then releasing outside the cell in the form of exosomes [15]. Regarding exosome budding, the most accepted hypothesis is the endosomal sorting complex required for transport (ESCRT) family catalytic [16]. However, exosome biogenesis is not substantial reduction when the activity of the ESCRT family is inhibited [17], and researchers have discovered ESCRT-independent mechanisms affecting the production of exosomes, such as heterogeneous nuclear ribonucleoprotein-dependent pathway, neutral sphingomyelinase 2-dependent pathway, and a recently discovered novel pathway marked by RAB31 [18, 19]. ESCRT-dependent and independent secretory pathways are shown in Figure S1 [20]. With the deepening understanding of exosome biogenesis, other mechanisms may be discovered in the future.

Exosomes are lipid bilayer membrane vesicles ~ 30–150 nm in diameter and 1.13–1.21 g/ml in density [21]. Almost all human cells can secrete exosomes which widely exist in various body fluids, including blood, urine, semen, cerebrospinal fluid, tears, saliva, breast milk, bile, ascites, lymph, and amniotic fluid [22]. Other EVs mainly include microvesicles and apoptotic bodies. Microvesicles also referred to as exosomes, derive from the plasma membrane and are 100–1000 nm in size. Studies have shown that microvesicles have largely similar functions to exosomes [23]. Apoptotic bodies, also known as apoptosomes, are vesicles with a 500–5000 nm diameter and are released by cells undergoing apoptosis [24]. The function of EVs mainly relies on their loaded cargo, including proteins and mRNAs. The remaining components consist of long noncoding RNAs (lncRNAs), microRNAs (miRNAs), DNAs, and circular RNAs (circRNAs) [25–30].

At present, it is hard to put forward specific and widely recognized markers of "exosomes" derived from MVB compared to other small EVs due to the lack of an optimal isolation approach and specific markers for EVs from different sources. In general, the term "exosomes" is used to refer to a heterogenous mixture of EVs that are less than 200 nm in diameter. Thus, it contains exosomal and non-exosomal particles [30–32]. According to the MISEV2018 guidelines, we substitute "small extracellular vesicles" for "exosomes" [9].

## Separation, detection and storage of sEVs

The premise of sEVs research is to isolate sEVs at high purity and in sufficient quantities. Whereas various separation methods have been systematically introduced in other studies [33, 34], we briefly summarize the methods in Table 1 which utilize density, size, membrane proteins, solubility, surface charge, and lipid membrane of sEVs and point out their merit and demerit in this review. Density Ultracentrifugation (UC) is the most widely used method for isolating sEVs with minimal reagents and can obtain large volumes. However, the heavy protein contamination and comparatively low throughput negatively impact further studies of these sEVs [35–38]. Density gradient ultracentrifugation (dgUC) is considered to be a gold standard, which can provide the highest purity of sEVs and enables researchers to isolate specific subpopulations. Despite this, the requirement of expensive equipment and rare recovery efficiency are significant disadvantages [39–41]. Acoustofluidics can maintain EVs integrity and require a minimal sample, but they may cause protein contamination [39, 42]. Solubility precipitation preserves sEVs integrity and has commercially available kits. However, the lowest purity has negative impacts on the downstream functional analyses [43–45]. Size ultrafiltration (UF) is easily integrated with other EVs isolation methods, but it is not as effective at producing pure samples as UC [46–48]. Immunoaffinity antibodies can provide very pure samples, but it is hard to harvest intact EVs from antibodies when downstream analyses require intact vesicles [49–51]. Moreover, charge dielectrophoresis allows for separating specific EVs subpopulations with good purity, but some disadvantages, including low yield and damaging EVs membranes, negatively impact the downstream analyses [52–54]. Size exclusion chromatography (SEC) isolates EVs via utilizing size, and the purity of separated EVs is equivalent to that of dgUC. However, SEC is not able to utilize to concentrate EVs samples. Aiming at this problem, researchers obviously improve the purification efficiency of EVs by combining SEC with UF [55, 56]. In addition, electrostatic interaction and lipid nanoprobes (LNP) utilize surface charges and their lipid bilayer to isolate EVs [57, 58]. These approaches can obtain EVs within a short time without ponderous equipment and allow to perform of downstream analyses and functional studies. However, protein contamination may influence the accuracy of downstream analysis [59]. In brief, it is impractical to isolate sEVs at present, but the combined application of various methods is still recommended.

**Table 1.** sEVs separation methods

Separation methods	Advantages	Disadvantages	References
Density Ultracentrifugation (UC)	The most widely used method for isolating sEVs with minimal reagents and can obtain large volumes	The heavy protein contamination and comparatively low throughput	[35–38]
Density gradient ultracentrifugation (dgUC)	It is considered to be a gold standard, which can provide the highest purity of sEVs and enables researchers to isolate specific subpopulations	The requirement of expensive equipment and rare recovery efficiency are significant disadvantages	[39–41]
Acoustofluidics	It can maintain EVs integrity and require a minimal sample	They may cause protein contamination	[39, 42]
Solubility precipitation	Solubility precipitation preserves sEVs integrity and has commercially available kits	The lowest purity has negative impacts on the downstream functional analyses	[43–45]
Size ultrafiltration (UF)	Size ultrafiltration (UF) is easily integrated with other EVs isolation methods	It is not as effective at producing pure samples as UC	[46–48]
Immunoaffinity antibodies	Immunoaffinity antibodies can provide very pure samples	It is hard to harvest intact EVs from antibodies when downstream analyses require intact vesicles	[49–51]
Charge dielectrophoresis	Charge dielectrophoresis allows for separating specific EVs subpopulations with good purity	Some disadvantages, including low yield and damaging EVs membranes, negatively impact the downstream analyses	[52–54]
Size exclusion chromatography (SEC)	Size exclusion chromatography (SEC) isolates EVs via utilizing size, and the purity of separated EVs is equivalent to that of dgUC	SEC is not able to utilize to concentrate EVs samples	[55, 56]
Electrostatic interaction and lipid nanoprobes (LNP)	It can obtain EVs within a short time without ponderous equipment and allow to perform of downstream analyses and functional studies	Protein contamination may influence the accuracy of downstream analysis	[57–59]

Besides, it is also required to develop more efficient and economical methods to detect sEVs. Rapid advances in on-chip detection technologies make sEVs easier to discover. We briefly summarize the sEVs detection methods in Table 2 and point out their advantages and disadvantages. The ELISA-based on-chip detection method is compassionate and provides noticeable color variations to indicate the presence of the target with 2–4 h to finish detection [60, 61]. The lateral flow immunoassays (LFIA) method, Au-conjugated anti-CD9 is used as the capture line. Meanwhile, Au-conjugated anti-CD63 is used for the detection/control line. It is less sensitive than ELISA and takes about 2 h [62, 63]. In addition, several biosensors with fast and sensitive detection performance have been examined, including plasmon resonance and quantum dots (QDs), the readouts of which have compatibility with clinical settings [64–67]. Surface plasmon resonance (SPR), relying on light to excite electrons and then producing resonant oscillation currents, can be used to detect nanoparticles, including sEVs [68]. In 2018, Liu et al. developed a small SPR system to detect sEVs from lung cancer [65]. Moreover, as inorganic colloid tracers which are utilized in signal transduction labeling, QDs provide a sensitive way to detect sEVs [66]. In the future, sEVs may replace the current diagnostics, but before that, much work, including high specificity, low cost, and fast detection, needs to be fulfilled.

**Table 2.** sEVs detection methods

Detection methods	Advantages	Disadvantages	References
ELISA	High sensitivity, high specificity, simple operation	Long time consumption, single point testing	[60, 61]
Lateral flow immunoassays (LFIA)	Fast, relatively easy to operate, portable	Low sensitivity and specificity, poor quantitative performance	[62, 63]
Surface plasmon resonance(SPR)	High sensitivity, high specificity, real-time monitoring	High equipment cost, complex sample processing	[65, 68]
Quantum dots (QDs)	Minimally invasive, fast and sensitive	It takes a long time and requires special equipment	[64–67]

Until now, there has been no consensus on the storage of sEVs. In the study of Rosario Maroto et al., it was pointed out that repeated freezing-thawed processes are extremely destructive to sEVs structure and physical properties, and that sEVs stored at + 4 °C and -80 °C are different, and their analysis found that 756 (89%) proteins did not change in abundance due to changes in storage temperature. However, after storage at + 4 °C, 61 proteins were depleted; In contrast, 31 proteins were more abundant in exosomes stored at + 4 °C than in sEVs stored at -80 °C, suggesting that small amounts of sEVs proteins are more sensitive to storage temperature. Moreover, according to the different storage conditions, a small proteome appeared in the supernatant, 67 proteins were enriched in the sEVs supernatant stored at -80° C, and 78 unique proteins were enriched in the sEVs supernatant stored at + 4 °C. These proteins include cytokines (CXCL15, CC10) and serine proteases (Serpina1c and -1d). Another finding of the study was that storage conditions affect the proteomic content of airway sEVs. Analysis of sEVs in cell culture showed that storage at + 4 °C had a significant effect on CD63 and Hsp70 content, a finding that confirmed the relative depletion of CD63 in stored samples [69].

## Biofunctions of sEVs in glioma

**Fig. 2:**
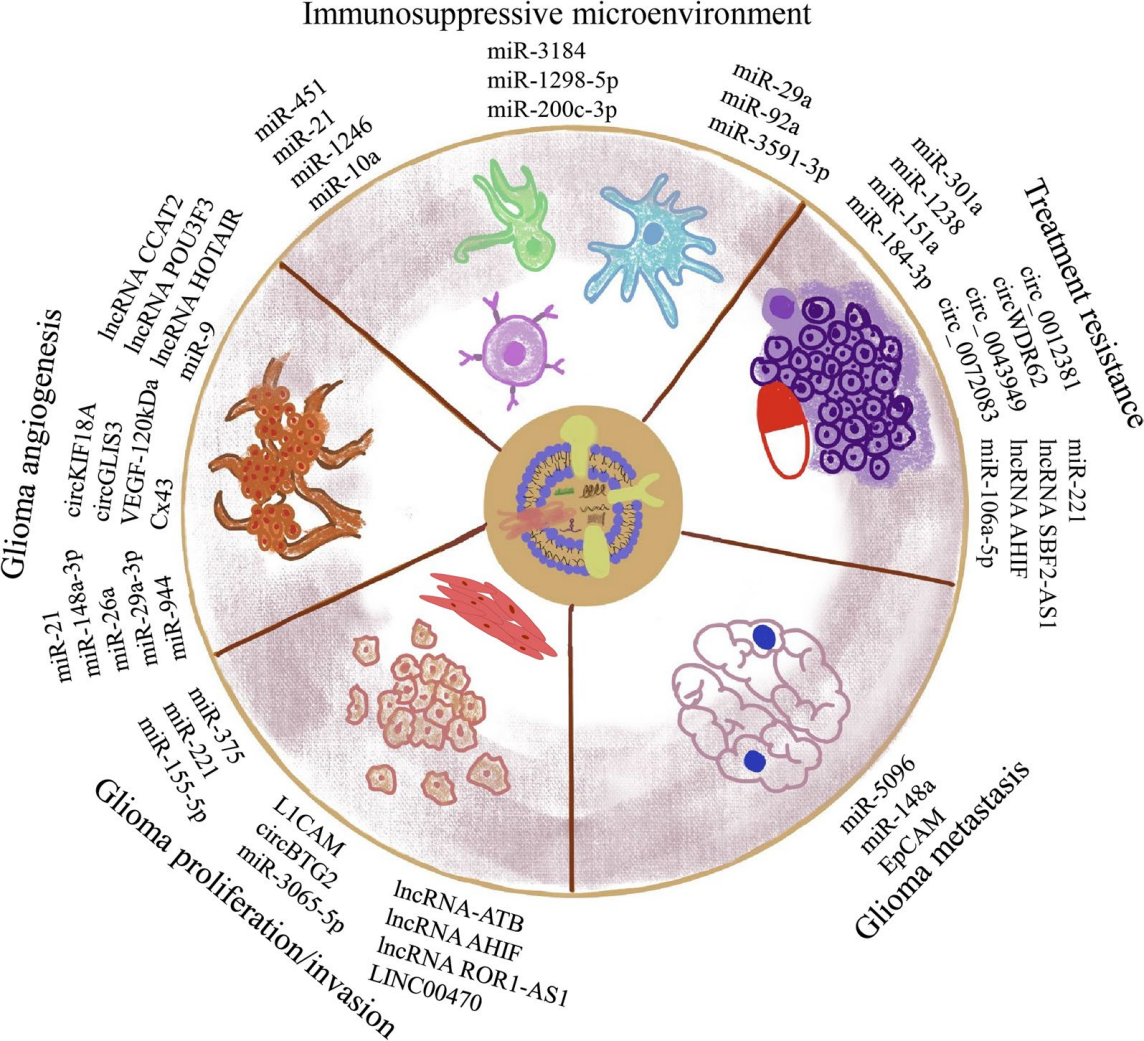
sEVs play vital roles in mediating glioma proliferation/invasion, angiogenesis, metastasis, immunosuppressive microenvironment, and treatment resistance

Numerous shreds of evidence have shown that sEVs mediate the occurrence and progression of various tumors by promoting intercellular communication, pro-inflammatory responses, and regulating the TME. At the same time, the function of sEVs in glioma has not been systematically reviewed. Here, we comprehensively show the biological function of sEVs in glioma (Fig. 2).

### sEVs and cell proliferation/invasion in glioma

The most essential characteristics of malignant glioma are rapid proliferation and extensive invasion, which involve complex molecular regulation and dynamic crosstalk between the tumor and microenvironment. This leads to difficulty in complete surgical resection and postoperative recurrence [70]. Therefore, the identification of critical molecules and related mechanisms involved in the proliferation and invasion of tumors is necessary for the development of new therapeutic strategies. Glioma-derived sEVs have been reported to play an essential role in tumor proliferation and invasion by regulating intercellular communication in local and distant microenvironments. Bian et al. demonstrated that sEVs derived from glioma cells and transporting lncRNA-ATB can activate astrocytes via inhibition of miR-204-3p, leading to promotion of glioma cell invasion by the activated astrocytes [71]. LncRNA AHIF, the natural antisense transcript of hypoxia-inducible factor 1α (HIF 1α), has been reported to induce glioma cell proliferation, invasion, and radioresistance through sEVs, suggesting its potential as a therapeutic target [72–74]. Furthermore, Chai and colleagues have found that glioma tissues exhibit upregulated expression of lncRNA ROR1-AS1 in comparison to normal tissue, with high levels of lncRNA ROR1-AS1 indicating a poor prognosis. Mechanistic investigations have revealed that sEVs carrying lncRNA ROR1-AS1 derived from glioma cells can facilitate glioma progression by suppressing miR-4686 [75]. Ma et al. revealed that LINC00470 was up-regulated in serum sEVs from glioma patients and correlated with disease progression and postoperative survival of glioma patients, and sEVs-LINC00470 in GBM can bind to miR-580-3p in glioma cells to regulate WEE1 expression and activate the PI3K/AKT/mTOR pathway, thereby enhancing the proliferation of glioma cells [76].

In addition to lncRNAs, sEVs loading miRNAs or proteins are also crucial contributors to glioma progression. Xu and colleagues reported that sEVs-miR-375 derived from glioma cells was able to activate the CTGF-EGFR oncogenic pathway to promote glioma proliferation and invasion [77]. Research by Yang et al. found that sEVs-miR-221 induced tumor proliferation and migration in glioma via targeting DNM3 [78]. The latest research have shown that glioma stem cells (GSCs)-derived sEVs contribute importantly to the plasticity, heterogeneity, and aggressiveness of glioma [79]. GSCs-derived sEVs-miR-155-5p slao play a key role in enhancing the invasiveness of glioma cells by targeting ACOT12 and promoting mesenchymal transformation [80]. In the study of Li et al., sEVs- miR-3065-5p derived from GSCs induced astrocyte transformation through the miR-3065-5p/DLG2 signal transduction axis, further promoting the tumorigenesis of GSCs [81]. Immunoglobulin κ J region recombinant signal binding protein (RBP-J) is a transcriptional regulator indicating that sEVs-circBTG2 secreted by RBP-J overexpressed macrophages inhibits the proliferation and invasion of glioma cells through the circBTG2/miR-25-3p/PTEN pathway [82]. L1-cell adhesion molecule (L1CAM), the autocrine/paracrine of which is one of the factors that promote glioma cell proliferation, migration, and invasiveness [83, 84]. In another study, researchers isolated tumor cell sEVs packaging L1CAM and found these sEVs promoted glioma cell migration and proliferation. Furthermore, the results of in vivo experiment, which employed the white leghorn chicken embryo, were in accordance with in vitro experiment [85].

### sEVs and glioma angiogenesis

Continuous growth is one of the main characteristics of malignant tumors. Angiogenesis being able to provide nutrition and oxygen, is an important factor in the sustained growth of most solid tumors [86]. Similarly, GBM, which has the worst prognosis, has been demonstrated to be the most vascularized tumor [87, 88]. Recently, many studies indicated that sEVs are involved in glioma angiogenesis. Lang and colleagues found that sEVs derived from glioma cells transferred lncRNA CCAT2 to endothelial cells. The angiogenesis-related genes, including VEGF, TGFβ, FGF, and KDR, were then activated to promote angiogenesis [89]. Another study reported that sEVs delivering lncRNA POU3F3 could promote glioma angiogenesis [90]. In addition, the glioma cells derived sEVs which packaged lncRNA HOTAIR, and miR-9, can be transferred to endothelial cells, both leading to increased angiogenesis [91, 92]. Other reports indicate that M2 polarized GBM-related microglia-derived sEV-circKIF18A participate in GBM angiogenesis by targeting FOXC 2 and that glioma secretion of circGLIS3-loaded sEVs can also induce endothelial cell angiogenesis [93, 94]. As a member of the scaffolding adaptor protein family, ERBB receptor feedback inhibitor 1 (ERRFI1) plays a vital role in the epidermal growth factor receptor (EGFR) signaling pathway [95]. Wang et al. reported that sEVs-miR-148a-3p secreted by glioma cells stimulated glioma angiogenesis via activating the EGFR/MAPK signaling pathway by ERRFI1 inhibition [96]. In GBM, increased miR-21 has the function of upregulating VEGF expression. Sun and colleagues found that GSCs-derived sEVs promoted the angiogenesis of endothelial cells via miR-21/VEGF/VEGFR2 signaling [97]. Moreover, sEVs-miR-26a, which were derived from GSCs, could also stimulate the angiogenesis of microvessel endothelial cells in glioma by targeting PTEN and further activating the PI3K/Akt signaling pathway [98]. In addition, GSCs-derived sEVs-miR-944 inhibited glioma progression and angiogenesis by inhibition of VEGFC expression and inhibition of AKT/ERK signaling pathway [99]. Unlike the above, Zhang et al. reported that sEVs-miR-29a-3p derived from engineered human mesenchymal stem cells suppressed glioma angiogenesis [100].

Wang and colleagues found a unique form of VEGF with a length of 120-kDa (VEGF-120 kDa) existing in sEVs which were derived from GBM cells. The further study verified that the VEGF-120 kDa was a specific isoform of VEGF-C. In addition, by binding to VEGF receptor 2 (VEGFR2) and then inhibiting the Hippo pathway, the VEGF-120 kDa derived from GBM cells strongly stimulated the expression of tafazzin (TAZ) in endothelial cells, which eventually promoted angiogenesis [101]. Another study showed that hypoxia increased connexin 43 (Cx43) levels in sEVs secreted by glioma cells and acted on vascular endothelial cells to promote glioma angiogenesis [102].

### sEVs and glioma metastasis

In many cancer-related deaths, metastasis is an important issue [103]. As one of the most dangerous cancers, GBM rarely occurs in distant metastasis but mainly spreads through local invasion of the brain. However, GBM can spread to the CNS, which makes it hard to completely remove the tumor by surgery [104]. Emerging evidence suggests that advanced cancer cells can secrete sEVs that facilitate tumor progression [105, 106]. Recently, a study revealed that miR-5096 was able to stimulate the formation of filopodia filamentous pseudopodia and promote glioma cell invasion via regulating the K^+^ channel Kir 4.1. Further studies showed that miR-5096 could also promote the secretion of sEVs leading to GBM metastasis [107]. Cell adhesion molecule 1 (CADM1), a neural tissue-specific protein, plays an important role in cell–cell adhesion and is able to suppress the activation of STAT3 signaling, which is usually activated in GBM [108–110]. In addition, suppressing the phosphorylation of STAT3 can obviously decrease metastasis [111], and it has been reported that miR-148a could facilitate GBM progression via increasing CADM1/STAT3 signaling [112, 113]. Cai and colleagues found that miR-148a loaded in sEVs could promote GBM progression and metastasis by activating STAT3 signaling via CADM1, and the level of which in body fluids of GBM patients was higher than that of healthy individuals. These suggest that sEVs-miR-148a may serve as an effective diagnostic biomarker for GBM [114]. As a member of the adhesion molecules family, epithelial cell adhesion molecule (EpCAM) is a single transmembrane protein encoded by the tumor-associated calcium signal transduction gene 1 (TACSTD1) [115]. The research of Gu and colleagues showed that sEVs-EpCAM promoted glioma metastasis via targeting CD44 signaling molecules which are on the surface of glioma cells [116].

### sEVs in the TME of glioma

In gliomas, TME consists of non-tumor cells, including microglia, resident astrocytes, endothelial cells, extracellular matrix components, tumor-associated macrophages (TAMs), proteins, and secreted molecules. All these components play important roles in intercellular communication with tumor cells, thereby regulating disease progression [117]. GBM establishes a highly immunosuppressive microenvironment, promotes tumor progression, and has a typical characteristic of inflammatory response with an accumulation of macrophages via communicating with normal brain cells [118, 119]. GBM can recruit immune cells from the bloodstream, with monocytes comprising the predominant subset. Monocytes are highly malleable and classified into M1 macrophages with the function of pro-inflammatory and M2 macrophages with the function of anti-inflammatory [120, 121]. It has been reported that microglia/macrophages account for up to one-third of the tumor mass. Moreover, glioma-associated microglia and macrophages have been identified as key players in the resistance to immunoregulatory therapy [122, 123]. Therefore, the targeting of these immune cells and related molecules offers a novel and promising therapeutic strategy for glioma patients.

Gabrusiewicz et al. demonstrated that macrophages convert to an immunosuppressed M2 phenotype after uptake by GSCs-derived sEVs, which may be able to act as an effective regulator of the immunosuppressive tumor microenvironment [124]. Similarly, Juliana Azambuja et al. also found the transformation of the macrophage phenotype into type M2 after being exposed to sEVs derived from GBM cell lines in culture, and further experiments in mice yielded the same results [118]. Van der Vos et al. investigated the relationship between sEVs-miRNA secreted by glioma cells and microglia. They found that miR-451/miR-21 in sEVs derived from glioma cells were transported to microglia, resulting in increased proliferation of microglia and the transfer of cytokines profile to immunosuppression [125]. Glioma cells have been shown to secrete sEVs loaded with miR-3591-3p and target TAMs to promote the establishment of an immunosuppressive microenvironment [126]. In addition, research by Li et al. found that sEVs-miR1246 derived from hypoxic glioma could regulate NF-κB and STAT3 pathways by targeting TERF2IP to induce polarization of M2 macrophages and promote the proliferation and metastasis of gliomas [127]. Similarly, glioma-derived sEVs-miR-3184 were able to polarize macrophages to an M2-like phenotype and exacerbate tumor progression [128]. In the latest study, Guo et al. found that hypoxia drove GSCs to produce higher levels of glutamate, which activated local neurons. Neuronal activity promoted GBM progression by facilitating microglial M2 polarization through enriching miR-200c-3p in neuron-derived sEVs [129].

Myeloid-derived suppressor cells (MDSCs) were reported to play a pivotal role in regulating the formation of immunosuppressive environments that enable gliomas to evade host immune responses. However, the exact mechanism is not yet clear. Guo et al. observed that hypoxia-stimulated glioma-derived sEVs-miR-10a and miR-21 mediated differentiation and activation of MDSC, which made a stronger ability to induce MDSCs than normoxia-stimulated glioma-derived sEVs [130]. Another research by the same authors showed that glioma-derived sEVs-miR-29a/miR-92a could also promote the generation of the immunosuppressive microenvironment by stimulating the proliferation and differentiation of functional MDSCs [131]. In addition, sEVs-miR-1298-5p in cerebrospinal fluid (CSF) can promote the immunosuppressive effect of MDSCs, and then promote the development of glioma [132].

### sEVs and resistance to treatment

Maximum surgical resection followed by chemotherapy and radiotherapy is the standard treatment for high-grade gliomas. However, GBM, the highest glioma grade, is often resistant to multiple treatments [133, 134]. The resistance of glioma to radiotherapy and chemotherapy is the main factor affecting the treatment effect and leading to poor prognosis. Therefore, it is necessary to figure out the underlying mechanism and explore new treatments to reverse the resistance of GBM to radiation and cytotoxic drugs. In recent years, accumulating shreds of evidence reveals that sEVs, playing a vital role in cell–cell communication, may cause horizontal transmission of resistance capacity between cancer cells [28, 135].

Research by Yue et al. showed that sEVs-miR-301a derived from hypoxic GBM cells could be transported to corresponding normoxia-cultured cells, leading to radiation resistance via directly targeting TCEAL7 genes. This may provide a new target to reverse the resistance of glioma cells to radiotherapy [136]. In Guo et al. 's study, neuronal activation led to increased miR-184-3p content in sEVs, which were transmitted to GSCs and decreased N6-methyladenosine (m6A) level in GSCs by inhibiting the expression of RBM15. RBM15 deficiency decreased m6A modification of DLG3 mRNA and subsequently induced GSC proneural-to-mesenchymal transition by activating the STAT3 pathway to support glioblastoma progression and radioresistance [137]. Zhang et al. reported an upregulation of Circ_0012381 expression in radiation-treated GBM cells, and Circ_0012381 entered microglia through sEVs and induced M2 type microglia to increase ARG 1 expression, and further promoted the growth of GBM cells after radiation treatment. Therefore, inhibition of sEVs secretion may represent a promising approach to improve the therapeutic outcome of radiotherapy in GBM patients [138]. Temozolomide (TMZ), a monofunctional DNA-alkylating agent, acts as the first-line chemotherapy drug for the treatment of glioma [139]. It was reported that sEVs-miR-106a-5p derived from hypoxic glioma cells decreased the sensitivity of glioma cells to TMZ chemotherapy through the downregulation of PTEN [140]. Yang and colleagues revealed that sEVs-miR-221 induced TMZ resistance in glioma via targeting DNM3 [78]. TMZ resistance greatly reduces the effectiveness of treatment. Moreover, related studies have shown that sEVs secreted by TMZ-resistant glioma cells can spread TMZ chemoresistance to TMZ-sensitive glioma cells. Yin et al. revealed that TMZ-resistant glioma cells secreted sEVs containing bioactive miR-1238, which could be absorbed by TMZ-sensitive cells, then acquire TMZ resistance. Thus, sEVs-miR-1238 may be an effective biomarker for assessing the effect of chemotherapy [141]. Zeng et al. reported that overexpression of miR-151a made GBM cells sensitive to TMZ by inhibiting the X-ray repair cross-complementing 4 (XRCC4), which can trigger the DNA repair. They further incubated GBM receptor cells with sEVs derived from TMZ-resistant or TMZ-sensitive cells. The results showed that GBM receptor cells co-cultured with sEVs secreted by TMZ-resistant glioma cells were more resistant to TMZ. However, when they restored miR-151a in TMZ-resistant sEVs, the TMZ resistance in GBM receptor cells was significantly reduced [142].

In addition to the miRNAs, the circWDR62 delivery mediated by sEVs can promote TMZ resistance and malignant progression in vitro and in vivo by targeting the glioma miR-37–30-3p/MGMT axis. In addition, sEVs-circWDR62 derived from serum may act as an effective prognostic marker for glioma [143]. The study of Ding et al. showed that sEVs-circ_0072083 level was enhanced in TMZ-resistant patients and indicated a lower overall survival in glioma. Mechanically, sEVs-circ_0072083 promoted TMZ resistance via increasing NANOG via regulating miR-1252-5p mediated degradation and demethylation in glioma [144]. Other studies have shown that TMZ-resistant GBM cell-derived sEVs-circ_0043949 promotes TMZ resistance through upregulation of ITGA1 expression, providing a potential therapeutic target for TMZ-resistant GBM [145]. Zhang et al. revealed that sEVs could deliver lncRNA SBF2-AS1 from TMZ-resistant glioma cells to TMZ-sensitive glioma cells to spread TMZ resistance in glioma. The mechanism is that lncRNA SBF2-AS1 functioned as a ceRNA and sponged miR-151a-3p to regulate the expression of XRCC4, thus accelerating the repairment of TMZ-induced DNA damage [146].

## Potential clinical applications of sEVs in glioma

### sEVs in glioma diagnosis: promising candidates for liquid biopsy

In the field of neuro-oncology, the diagnosis and monitoring of gliomas are still challenging [147]. The main methods currently include neuroimaging and histological analysis of brain biopsy samples. The imaging method is a non-invasive examination, which does little harm to patients but is not sensitive enough, especially in the early stage of the tumor. Tumor tissue biopsy is able to accurately diagnose and evaluate the development of tumors. However, it cannot be performed in large quantities and repeatedly due to the characteristic of invasion. In addition, biopsy specimens may not completely represent the entire tumor. Thus, it is necessary to develop a non-invasive and accurate method of evaluating tumors to improve the quality of life belonging to GBM patients. Liquid biopsy referring to a new method for assessing the progress of GBM tumors and monitoring the effects of treatment, is attracting more and more attention.

Although liquid biopsy has been proven to be promising, the development of clinically validated biomarkers for tumor detection remains a considerable challenge, especially for gliomas. Currently, the most researched biomarkers mainly include circulating tumor cells (CTCs), circulating tumor DNA (ctDNA), and EVs [148]. The advantage of CTCs is that they can analyze the entire tumor genome, but CTCs are difficult to detect due to their small quantity and can only present a single cell type of the heterogeneous tumor. In addition, although ctDNA seems to contain more mutations belonging to tumors than CTCs, it can still not reflect the heterogeneity of tumors. In comparison, sEVs containing many kinds of functional biomolecules can reveal the complicated heterogeneity of the entire tumor. More importantly, they are very stable and readily available in almost all types of human biological fluids [149]. In addition, due to nano-sized characteristics, sEVs can cross the intact blood–brain barrier (BBB), which has the function of protecting the central nervous system from toxins and infectious pathogens [150–152]. These factors make sEVs the most promising biomarker suitable for early diagnosis and genotyping of tumors, regardless of the stage.

As early as 2008, Johan Skog et al. successfully detected the status of EGFRvIII in GBM by using microvesicles extracted from peripheral blood. The researchers summarized that longitudinal blood sampling provided a novel approach to monitoring the genetic dynamics of tumors [153]. Fraser et al. found that the amount of sEVs-protein is correlated with the invasion of glioma, which reveals the potential of this measurement in glioma diagnosis [154]. In 2018, Manda et al. used sEVs as biomarkers to detect high-grade gliomas which were EGFR-positive [155]. In fact, 90% of GBM patients aberrantly express at least one of the four markers at the exosomal level, which are EGFR, EGRRvIII, podoplanin, and IDH1 [156]. So far, in the field of sEVs for tumor diagnosis, protein-loaded sEVs are the most frequently studied [157]. Nevertheless, other components that sEVs contain, such as miRNAs, have also attracted increasing attention.

Research by Yang et al. revealed that the expression level of miR-221 was elevated in high-grade glioma tissues, and sEVs-miR-221 derived from the serum of glioma patients had a higher level than that of the control group by further study. What's more, the level of sEVs-miR-221 in serum increases with the elevation of glioma grade. This research suggested that sEVs-miR-221 derived from serum may potentially serve as a valuable biomarker for glioma diagnosis [78]. More promising data came from Manterola et al. Firstly, they isolated sEVs from the serum of 30 GBM patients and 30 healthy people. Then, they found that two miRNA (miR-564-3p and miR-320) and one small noncoding RNA (RUN6–1) presented the greatest difference in expression via miRNA chip technology, and the further study found that their expression level was significantly correlated with the diagnosis of GBM patients. Moreover, RNU6-1 was consistently an independent predictor of GBM diagnosis [158]. In addition, a study by Lan et al. revealed that the level of sEVs-miR-301a derived from serum was significantly increased in glioma patients than that in healthy controls. Further research showed that heightened levels of serum sEVs-miR-301a were associated with increasing pathological grades of glioma. Notably, the levels of serum sEVs-miR-301a were obviously decreased after the primary tumor was surgically removed but subsequently increased during GBM recurrence. These findings revealed that serum sEVs-miR-301a might act as a valuable Diagnostic biomarker for glioma [159].

Cerebrospinal fluid (CSF) examination has been widely utilized in the clinical monitoring of CNS diseases, but it is rarely used in glioma so far. MiR-21, the expression level of which was up-regulated in glioma and associated with the histological grade of glioma [160, 161]. Shi and colleagues found that the levels of sEVs-miR-21 isolated from CSF of glioma patients were obviously higher compared to healthy subjects. In contrast, there was no difference in the expression of sEVs-miR-21 isolated from serum. Furthermore, the CSF-derived sEVs-miR-21 levels were associated with tumor spinal/ventricle metastasis and recurrence [160]. This suggested sEVs-miR-21 in CSF might be a promising diagnostic and prognostic biomarker for glioma patients.

There are some other reports of sEVs serving as promising diagnostic biomarkers [80, 114, 143, 146, 162–165]. We summarize in Table 3.

**Table 3.** sEVs serve as potential diagnostic biomarkers

Cargo	Source of sEVs	System	Biomarker potential	References
EGFR, EGRRvIII, podoplanin & IDH1	Blood	GBM	Diagnostic biomarker	[156]
Cavin1	Serum	GBM	Diagnostic & prognostic biomarker	[162]
lncRNA HOTAIR	Serum	GBM	Diagnostic biomarker	[163]
LncRNA SBF2-AS1	Serum	GBM	Diagnostic biomarker for therapy-refractory GBM	[146]
miR-148a	Serum	GBM	Diagnostic biomarker	[114]
miR-221	Serum	Glioma	Diagnostic biomarker	[78]
miR-320, miR-574-3p & RNU6-1	Serum	GBM	Diagnostic biomarker & tumorigenesis factors	[158]
miR-301a	Serum	Glioma	Diagnostic & prognostic biomarker	[159]
miR-21	CSF	Glioma	Diagnostic & prognostic biomarker	[160]
miR-21, miR-222 & miR-124-3p	Serum	Glioma	Diagnostic biomarker & grade prediction	[164]
miR-454-3p	Serum	Glioma	Diagnostic biomarker	[165]
circWDR62	Serum	Glioma	Prognostic biomarker	[143]
miR-155-5p	plasma	Glioma	Diagnostic biomarker & grade prediction	[80]

### The potential application of sEVs in anti-glioma therapy

In recent years, the function of sEVs in intercellular communication has become widely known. They can reveal much information about the parental cells, leading to in-depth studies on the diagnostic application of sEVs. In fact, sEVs also have great application potential in the therapy of cancer, although the current research is relatively backward. Current biological therapeutics, such as short interfering RNA and recombinant proteins, have many deficiencies, including easy degradation, restricted membrane permeability, and triggering undesirable immune reactions. However, sEVs have the following advantages as glioma treatment. First, sEVs can transport their contents to specific targets through their surface molecules and homing characteristics, which is good specificity. Second, the double-layer phospholipid membrane structure of sEVs protects its contents from the decomposition of protease and RNAase, which is highly stable. Third, self-derived sEVs have a high degree of histocompatibility and do not induce an adverse immune response, which is safe. Fourth, the clearance from the mononuclear phagocyte system is reduced due to their nanoscale size. Fifth, sEVs can cross the BBB [166].

There are two ways to load sEVs with cargo, including exogenous loading and endogenous loading. In endogenous loading, the modifications occur during the formation of the sEVs. In exogenous loading, sEVs are first isolated and then modified by freeze–thaw cycles, incubation, sonication, electroporation, and extrusion [167]. Researchers use sEVs to deliver tumor-suppressing ncRNAs for research. For example, in the rat model of GBM, Hamideh, and colleagues showed that the administration of sEVs loaded with miR-21-sponge construct could significantly reduce the tumor volume [168]. Katakowski et al. found that sEVs derived from MSCs overexpressing miR-146b decreased the growth of glioma xenograft in a rat model of primary brain tumor via intra-tumor injection [169]. Similar research was performed by Fareh and colleagues. They engineered primary glioma cells to stably express the miR-302–367, which has the function of inhibiting tumors and was mainly packaged in sEVs. These sEVs were taken up by neighboring GBM cells leading to antitumor effects both in vivo and in vitro [170]. Moreover, Munoz et al. revealed that anti-miR-9-delivering sEVs improved the expression level of multidrug transporters and the sensitivity to TMZ in drug-resistant GBM cells, resulting in higher cell mortality and caspase activity. This may be an effective way to overcome the resistance of gliomas to chemotherapy [171]. Qian et al. used sEVs derived from neural stem cells to deliver miR-124-3p to glioma and significantly inhibited the malignant biological behavior of glioma cells [172]. Another approach is to package the drug itself into sEVs. It has been reported that paclitaxel-loaded sEVs resulted in a nearly 50-fold increase in cytotoxicity in multidrug-resistant tumors compared to paclitaxel without exosomes [173]. In addition, the researchers developed a nano delivery system based on functionalized macrophage sEVs targeted with heliostat and PPM1D-siRNA for PPM1D mutant diffuse intrinsic pontine glioma, with higher administration efficiency and better therapeutic efficacy than free drugs [174]. Other reports on the use of dual receptor-specific sEVs as carriers loaded with TMZ and O6-benzyguanine for the eradication of TMZ-resistant GBM showed that the sEVs had good proliferation inhibition in vitro and prolonged the median survival of U87MG tumor-bearing mice without causing adverse effects [175]. The latest approach is achieved by using a microfluidic electroporation approach in which a combination of nano- and milli-second pulses produces large amounts of IFN-γ mRNA-loaded sEVs with CD64 overexpressed on their surface. The CD64 molecule serves as an adaptor to dock targeting ligands, such as anti-CD71 and anti-programmed cell death-ligand 1 (PD-L1) antibodies. The resulting immunogenic sEVs preferentially target glioma cells and generate potent antitumour activities in vivo, including against tumours intrinsically resistant to immunotherapy [176]. These studies demonstrate the potential value of sEVs in the treatment of gliomas.

## Conclusions and prospects

Gliomas present poor prognosis due to invasion and resistance to multiple treatments. Up to now, the complex pathogenesis of glioma is still not fully understood. sEVs can transport a variety of biomolecules and mediate communication between tumor cells and TME via the horizontal transfer of information. In gliomas, extensive studies have shown that sEVs are selectively packaged, secreted, and transferred between cells, thus regulating multiple biological characteristics, including proliferation, invasion, angiogenesis, immune escape, and treatment resistance. Moreover, the ability to easily cross various biological barriers (such as BBB) provides a broad prospect for clinical applications of sEVs in the diagnosis and treatment of gliomas.

Nevertheless, there are still many challenges in the view of clinical applications. Firstly, the separation and purification of sEVs, which is the premise for large-scale use of sEVs, have not been standardized. Secondly, more reliable biomarkers should be identified. In the future, a diagnostic panel will combine multiple biomarkers rather than single biomarkers to provide information on early diagnosis and prognosis. Then, how to maximize the use, standardization, and quantification of sEVs still needs further research due to its limited genetic information. Although the therapeutic applications of sEVs have made great progress in vitro and animal experiments, before applying sEVs to the clinical treatment of glioma, sufficient safety, targeting efficacy, and avoidance of adverse reactions should be considered ensured. In addition, recent studies have also found that neuronal sEVs play a key role in the malignant biological behaviors of glioma such as radiotherapy resistance and immunosuppression, which is worthy of further in-depth and extensive study. Finally, more structured studies in different laboratories around the world and achieving a consensus on sEVs terminology are needed to advance the field of sEVs.

We believe that with the improvement of technology, sEVs will be widely used in the clinical diagnosis, treatment, and prognosis of glioma in the near future.

### Supplementary Information

**Additional file 1: Figure S1**. The ESCRT- dependent and independent pathway are implicated in controlling the cargos sorting of exosomes. [20] Copyright 2020, Molecular Cancer.

## Data Availability

No datasets were generated or analysed during the current study.
