# Supplementary figures and images for: The role and application of small extracellular vesicles in glioma
Source: Cancer Cell Int. 2024 Jun 29;24:229. doi: 10.1186/s12935-024-03389-z (PMC11218314; doi:10.1186/s12935-024-03389-z)

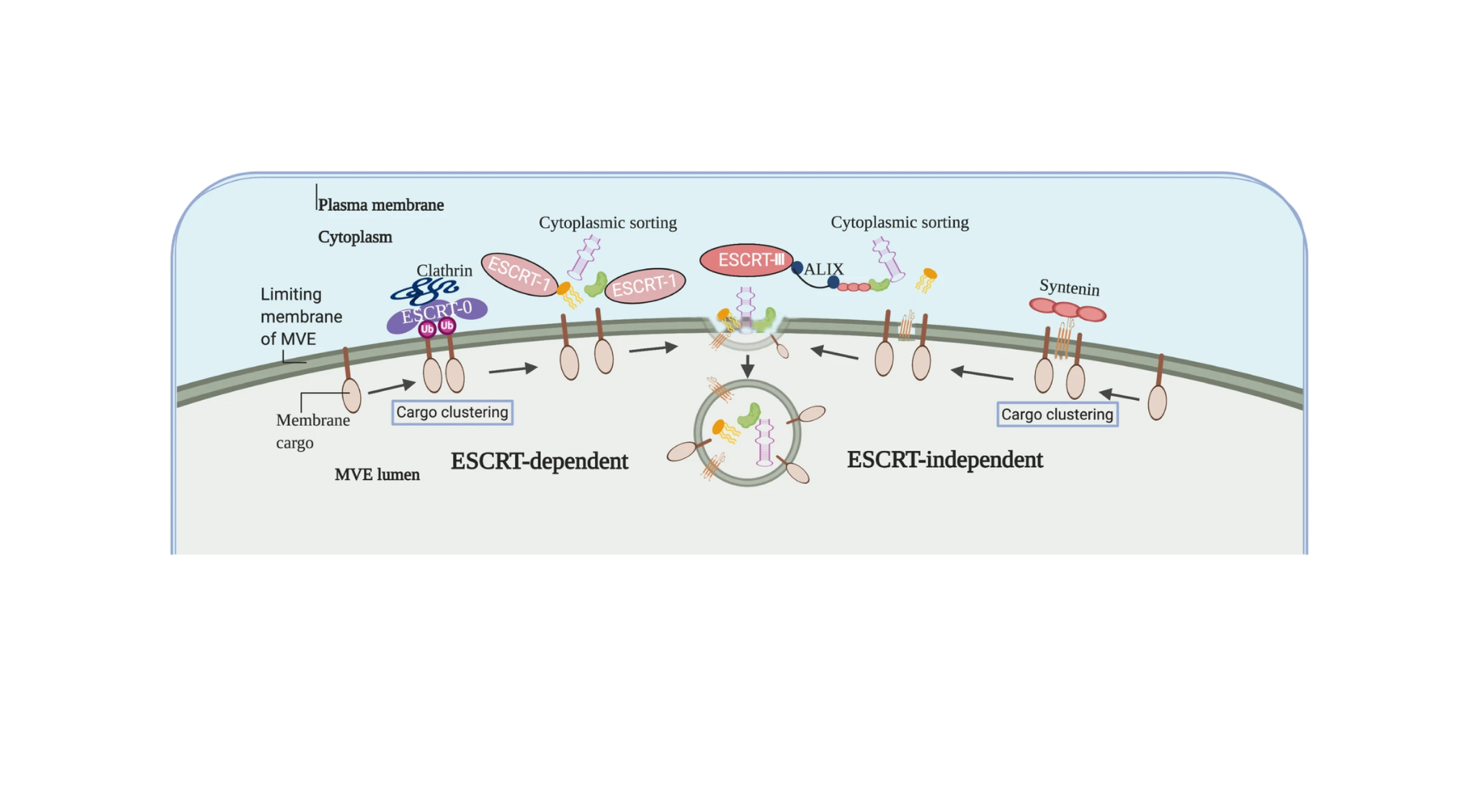

Supplement: Supplementary file 1 — Additional file 1: Figure S1. The ESCRT- dependent and independent pathway are implicated in controlling the cargos sorting of exosomes. [20] Copyright 2020, Molecular Cancer. [file 12935_2024_3389_MOESM1_ESM.tif]
